# Supplementary material for: Identification and Expression Analysis of the SKP1-Like Gene Family under Phytohormone and Abiotic Stresses in Apple (Malus domestica)
Source: Int J Mol Sci. 2023 Nov 16;24(22):16414. doi: 10.3390/ijms242216414 (PMC10671573; doi:10.3390/ijms242216414)
Supplement: Supplementary file 1 [file ijms-24-16414-s001.zip › Supplementary Table S1.pdf]

Supplementary Table S1 Physical and chemical properties of MdSKP1-like protein in apple

Name	Gene accession No.	Size / aa	Molecular weight / D	Isoelectric point	Formula	Instability index	Aliphatic index	Grand average of hydropathicity	Hydrophilicity
MdSKP1-Like 1	MDP0000193231	108	12147	4.62	$C_{530}H_{831}N_{145}O_{168}S_7$	34.73	85.65	-0.313	Hydropathicity
MdSKP1-Like 2	MDP0000278773	356	41130	5.21	$C_{1776}H_{2857}N_{511}O_{570}S_{20}$	55.94	76.46	-0.782	Hydropathicity
MdSKP1-Like 3	MDP0000263930	145	16443	4.99	$C_{737}H_{1188}N_{188}O_{223}S_6$	40.82	110.14	-0.019	Hydropathicity
MdSKP1-Like 4	MDP0000282839	355	40542	5.66	$C_{1742}H_{2852}N_{518}O_{565}S_{14}$	56.98	79.66	-0.824	Hydropathicity
MdSKP1-Like 5	MDP0000924440	156	17728	4.54	$C_{777}H_{1236}N_{202}O_{258}S_6$	50.05	84.42	-0.536	Hydropathicity
MdSKP1-Like 6	MDP0000177274	155	17525	5.09	$C_{763}H_{1235}N_{203}O_{239}S_{14}$	45.52	87.48	-0.264	Hydropathicity
MdSKP1-Like 7	MDP0000273940	165	18687	4.52	$C_{813}H_{1294}N_{216}O_{271}S_8$	39.15	81.58	-0.536	Hydropathicity
MdSKP1-Like 8	MDP0000306021	156	17769	4.61	$C_{779}H_{1243}N_{205}O_{254}S_7$	41.98	84.42	-0.481	Hydropathicity
MdSKP1-Like 9	MDP0000656961	135	15545	4.75	$C_{684}H_{1089}N_{181}O_{215}S_8$	37.34	90.37	-0.421	Hydropathicity
MdSKP1-Like10	MDP0000150245	190	21887	4.38	$C_{950}H_{1489}N_{249}O_{327}S_8$	43.28	70.74	-0.729	Hydropathicity
MdSKP1-Like11	MDP0000505840	163	18482	4.63	$C_{808}H_{1290}N_{214}O_{266}S_7$	42.25	83.80	-0.540	Hydropathicity
MdSKP1-Like12	MDP0000135806	160	18008	4.58	$C_{781}H_{1252}N_{206}O_{260}S_{10}$	30.86	79.38	-0.517	Hydropathicity
MdSKP1-Like13	MDP0000549178	161	18142	4.67	$C_{788}H_{1259}N_{209}O_{260}S_{10}$	38.93	78.26	-0.497	Hydropathicity
MdSKP1-Like14	MDP0000190030	207	23297	4.61	$C_{1014}H_{1601}N_{273}O_{343}S_6$	37.82	76.33	-0.587	Hydropathicity
MdSKP1-Like15	MDP0000278340	156	17777	4.57	$C_{781}H_{1243}N_{205}O_{255}S_6$	42.87	84.42	-0.521	Hydropathicity

MdSKP1-Like16	MDP0000255887	155	17529	4.27	$C_{770}H_{1219}N_{195}O_{256}S_{7}$	54.29	93.16	-0.259	Hydropathicity
MdSKP1-Like17	MDP0000273484	156	17728	4.6	$C_{780}H_{1244}N_{200}O_{257}S_{6}$	46.18	84.42	-0.534	Hydropathicity
