# Supplementary material for: Identification and Expression Analysis of the SKP1-Like Gene Family under Phytohormone and Abiotic Stresses in Apple (Malus domestica)
Source: Int J Mol Sci. 2023 Nov 16;24(22):16414. doi: 10.3390/ijms242216414 (PMC10671573; doi:10.3390/ijms242216414)
Supplement: Supplementary file 1 [file ijms-24-16414-s001.zip › Supplementary Table S2.pdf]

Supplementary Table S2 qRT-PCR primers for expression analysis of *MdSKP1-like* gene in apple

Gene	（5′–3′） Forward primer	（5′–3′） Reverse primer
GADPH	*TTCTCGTTGAGGGCTATTCCA*	*CCACAGACTTCATCGGTGACA*
MdSKP1-Like 1	*GAAGAGGGAGCAGAGGGAGCAG*	*CAGAAGTCGGCAGCCAAGATCAG*
MdSKP1-Like 2	*GGCGGATTTAGGGAGCGAACAC*	*AAGCTCAACATTGCAGTACCGACTC*
MdSKP1-Like 3	*GATACCATTGCCAGAGGTCAACTCC*	*GACAAACTCAGCCTCCCACTCTTC*
MdSKP1-Like 4	*GATCTCACCAGCCGAGCACTTG*	*AGGTTCCAACTTCTCCTCCTCTGTG*
MdSKP1-Like 5	*GACCTGACATGCCAGACAGTTGC*	*ACGACGAACTTCCTCCTCTTCCTC*
MdSKP1-Like 6	*GAAAGGGATGGGAGCTTCCAAGAAC*	*GTTCGAGCGACCTGGTACTTGATG*
MdSKP1-Like 7	*CAACGTGACCAGCCACATCCTC*	*AGTGTTCTCGTCGCCAATCTTGC*
MdSKP1-Like 8	*GAGCTTGCTGGACCTGACTTGC*	*TGTTGAACGTCTTGCGAATCTCCTC*
MdSKP1-Like 9	*GGAGTCGCAGACCATCAAGCAC*	*GTAGCAATAGCCCTGTCCTCTTCAC*
MdSKP1-Like10	*CCTGGCCGCCGATTACTTGAAC*	*CGACGCCGAACAACTTCCTCAC*
MdSKP1-Like11	*AGATTCGCACGACCTTCAACATCAC*	*AAGCCCACTGGTTCTCCCTACG*
MdSKP1-Like12	*GATGAGGCGGTTGCTATGGAGTC*	*CGATGATCTTGGCGAGGATGTGG*
MdSKP1-Like13	*ACACCTGAACAGATTCGCACGAC*	*AAGCCCACTGGTTCTCCCTACG*
MdSKP1-Like14	*CAGACCGTCAAGTCGTTCTTCCAG*	*GCCTCATCGTGCTCCACTTTCC*
MdSKP1-Like15	*GAGCTTGCTGGACCTGACTTGC*	*TGTTGAACGTCTTGCGAATCTCCTC*
MdSKP1-Like16	*CGACAACTGTATTCCTTTGCCCAAC*	*CCACCTTTACGAACGCCTGATC*
MdSKP1-Like17	*GCCAGACGGTTGCAGACATGATC*	*ACGACGAACTTCCTCCTCTTCCTC*
