# Supplementary figures and images for: Identification and Expression Analysis of the SKP1-Like Gene Family under Phytohormone and Abiotic Stresses in Apple (Malus domestica)
Source: Int J Mol Sci. 2023 Nov 16;24(22):16414. doi: 10.3390/ijms242216414 (PMC10671573; doi:10.3390/ijms242216414)

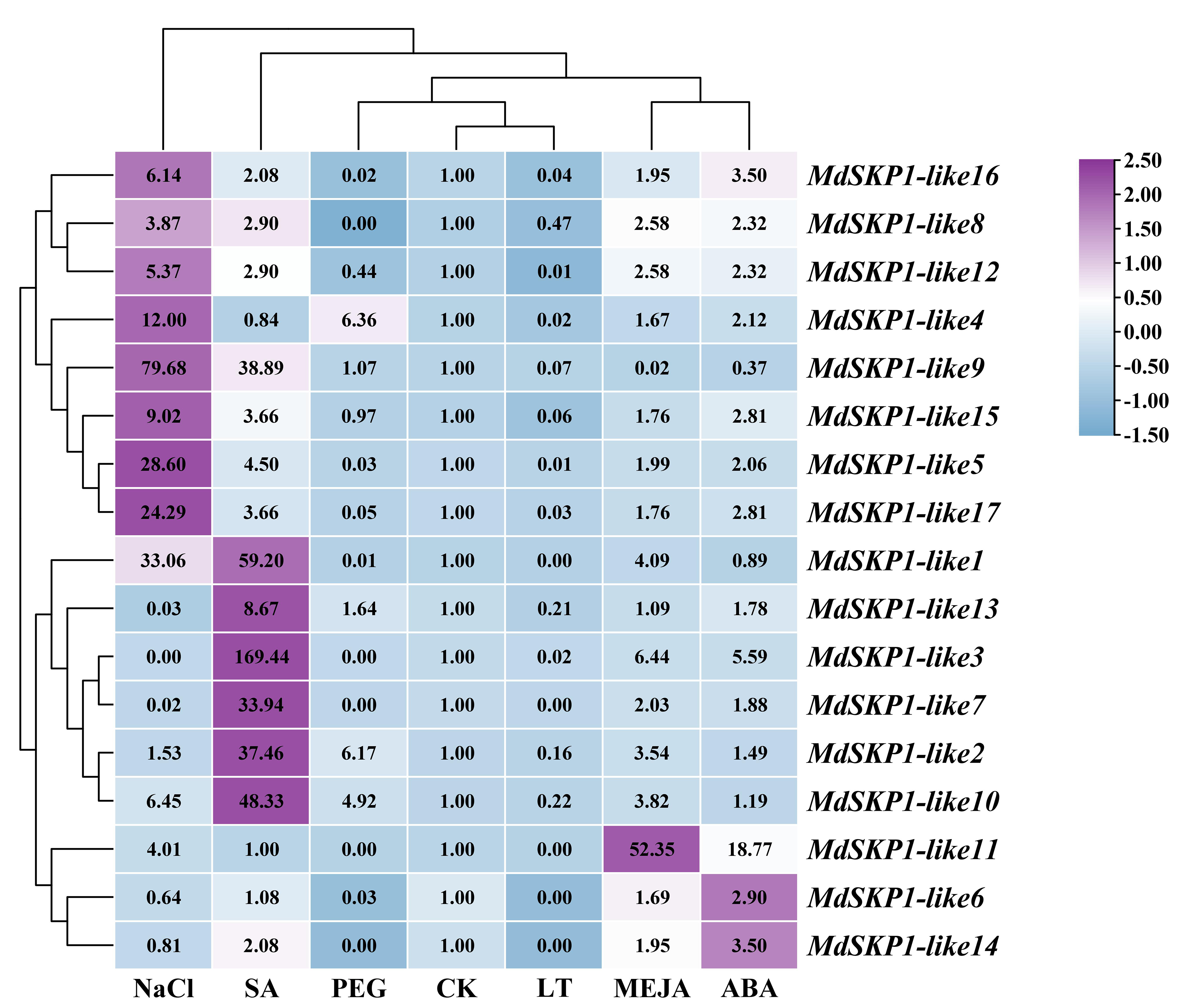

Supplement: Supplementary file 1 [file ijms-24-16414-s001.zip › Supplementary Figure S2.tif]
